# Hospital at home: emergence of a high-value model of care delivery

**DOI:** 10.1186/s43162-023-00206-3

**Published:** 2023-03-17

**Authors:** Sai Gautham Kanagala, Vasu Gupta, Sunita Kumawat, FNU Anamika, Brian McGillen, Rohit Jain

**Affiliations:** 1 Osmania Medical College, Hyderabad, India; 2 Dayanand Medical College and Hospital, Ludhiana, India; 3 Index Medical College Hospital & Research Center, Indore, India; 4 University College of Medical Sciences, Delhi, India; 5 Medicine, Penn State Health Milton S. Hershey Medical Center, Hershey, PA USA

**Keywords:** Hospital at home, Cost-effective, Patient satisfaction score

## Abstract

**Background:**

With increasing healthcare demands for acute illness in patients especially in the times of pandemic, healthcare organizations require modern solutions. Hospital at home (HaH) is one such tool that has the potential to solve these problems without compromising the care of the patients.

**Main body:**

Hospitals have been the conventional setting for managing acute sickness patients; however, it could be a very challenging environment for a few patients, especially for the older population who are highly susceptible to hospital-acquired infections. Health care in a hospital setting can also be very expensive, as it often involves a lot of healthcare professionals providing care. HaH service can provide the same quality of care expected in traditional settings.

**Conclusions:**

The median length of stay and the rate of readmissions were lower in people under HaH care. Compared with patients in a hospital setting, patients in HaH had better clinical outcomes. HaH unit provides an integrated, flexible, easy-to-scale platform that can be cost-effectively adapted to high-demand situations.

## Background

Hospital admission is despised by all. An innovative program called “hospital at home” (HaH) seeks to establish a bridge between the home and the hospital for better patient care. Hospital at home aims to provide hospital-level care in the comfort of the patient’s homes. Although this concept has been around for 25 years, its popularity is on the rise with increased hospitalizations due to COVID straining the existing inhospital infrastructure. The HaH program was first set up by Dr. John Bueron and Dr. Donna Regenstrief in 1995 [1]. In 1999, a study found that the HaH program was safe and effective in acutely ill elderly patients [2]. As the name suggests, HaH is an integrated system bringing care that is traditionally rendered inside of hospitals—daily physician or nurse visits (generally at least twice daily), monitoring vital signs, laboratory testing, intravenous fluids, and medication administration to patient homes, with the added provision of a hospital transfer, if the patient needs one [3]. Therefore, HaH provides an alternative venue for patients requiring inpatient care, which decompresses traditional hospitals to allow the sickest patients to be cared for there. The critical components of the HaH model are people, technology, processes, supply chain, and analytics. The evaluation of a patient takes place in the hospital, during urgent or proactive home visits, or over the phone. A physician or a rapid response team assesses a patient’s physical and psychological health, symptom distress, acute and chronic symptoms, functional status, illness stage, comorbidities, and motivation [4].

The physician at the emergency department assesses whether the patient requires inpatient hospitalization and the triage nurse screens for HaH care. Eligible patients are sent home after being examined by the attending physician and are provided with care at home, similar to the inpatient hospital setting. Before the patient is transferred home, a primary caregiver (typically a family member) is assessed for competence to provide prescribed care to the patient, and a case manager or social worker assesses the home environment to ensure it is suitable and safe for the patient. Typically, social inclusion criteria emphasize the home or dwelling with a roof, running water, Wi-Fi/Internet availability, and heating/air-conditioning systems. Consent is obtained from the patient, and they are transported by ambulance (along with biometric and communication devices needed to supervise care) to their home setting [5]. After the patient is transferred to the home, nursing care is started immediately to provide the preliminary portion of the admission and daily nursing visits according to the clinical need. All the patient care instructions are documented, and caregivers are taught to administer intravenous fluids and medications, and home nurses conduct tests, including ultrasounds, X-rays, and electrocardiograms (skills and ability vary by program). Physicians have daily visits with patients and are available 24/7 through telemedicine infrastructure to ensure that the patient’s condition is stable. The patient’s vitals, such as blood pressure, heart rate, electrocardiogram, pulse oximeter, and temperature, can be continuously monitored at home. Once the patient is stable enough to return to daily life activities, the care is transferred to the patient’s primary care physician [5].

## Main text

In HaH, the responsibilities of a physician vary extensively. The competencies and clinical abilities of a physician needed to administer HaH are determined by the program’s service delivery model, which includes admission avoidance and early discharge and the spectrum of clinical responsibilities that come with providing care. HaH physicians comprise a diverse group of clinicians, varying from community-based medical doctors to clinical specialists [6]. A HaH physician should be able to incorporate and organize care for patients allocated to them, including creating and reviewing clinical data; executing necessary tests, procedures, and treatments; and attending to all of the patient’s routine medical needs and management. This requires special training, similar to the hospitalist that provides inpatient care, with the added caveat that providers in the HaH model must be comfortable with providing this level of service without resources that normally exist with a simple mouse click. While HaH programs should, in theory, be able to deliver common therapies previously reserved for the inhospital setting—including intravenous fluid therapy, intravenous antibiotic therapy for the treatment of infectious diseases, intravenous antiviral therapy, intravenous corticosteroids, acute anticoagulation therapy, intravenous diuretic and inotropic therapy, intravenous blood product infusion, intravenous chemotherapy, oxygen therapy, simple radiology (e.g., abdominal and chest radiographs), ultrasound, venous Doppler, cardiac echocardiography, and even procedures such as thoracentesis and paracentesis—the inability to deliver these therapies or obtain studies in a near-immediate fashion can be overwhelming to the unprepared clinician [7].

Implementation of this program has been slow in the USA, but it is gaining momentum, especially due to the Covid-19 pandemic. The Acute Hospital Care at Home Program was created by the *Centers for Medicare* and *Medicaid Services* (CMS) to manage the overwhelming numbers of Covid-19-positive cases. According to the Centers for Medicare and Medicaid Services (CMS), more than sixty different acute conditions can be treated at home by following proper protocols [8]. In November 2020, CMS issued a waiver that allowed more flexibility to the hospitals in treating patients at home [8]. This led to a substantial expansion of the HaH program from twelve existing programs before the Covid-19 pandemic to 186 hospitals belonging to 66 different systems as of October 2021 [9]. In February 2022, CMS signaled a commitment to the long-term continuation of policies that would reimburse HaH programs, but these policies have not been formalized.

CMS has published a few compliance requirements for hospitals deploying HaH programs. A summary is shown in Table 1 below [9].

**Table 1:** CMS list of requirements for acute hospital-at-home program

Proper screening protocols
Daily evaluation either in-person or via telehealth by the physician
Twice a day visits daily by either a registered nurse or paramedics
Capability of immediately connecting to the attending nurse remotely
Responding to a decompensated patient within 30 min
Regularly tracking and reviewing patient safety metrics

There are two main types of HaH models currently in use, termed “direct” and “indirect” hospital at home. The “direct” HaH model provides acute hospital-level care in a patient’s home, rather than requiring admission to a hospital. Patients who require acute hospital care and are candidates for HaH (hospital at home) are typically identified in the emergency department or ambulatory environment and transported directly home to receive hospital-at-home care in this approach.

The second more “indirect” hospital-at-home model involves a brief overnight stay in the physical hospital for optimization, with discharge home and enrollment into the HaH program followed soon thereafter. Once inhospital optimization is complete, patients are returned to their homes and complete their treatment course outside of the hospital. Patients entering via this model may need to receive post-acute specialized rehabilitation assistance in the home after being discharged from the hospital [10].

The patients need to meet specific screening criteria to be eligible for enrollment in HaH. Elderly patients with chronic conditions and comorbidities with risks of iatrogenic consequences, functional decline, adverse medication responses, delirium, and falls are included. Patients are excluded if their acute sickness is severe enough (i.e., shock, ongoing acute myocardial infarction) to warrant the need for hospital-based services on an urgent/emergent basis. If the patient is in shock or experiencing a heart attack, they are not a good fit for the hospital at home and will need to be admitted to a hospital facility. This model considers patients who will not need MRIs, CT scans, and procedures (i.e., biopsy) and will have a shorter duration of hospital stay [11].

### Benefits

In this article, we write about the benefits and challenges faced by the HaH system stakeholders (patients, healthcare professionals, caregivers, and healthcare administrators) [9]. The primary benefactor of this model is the patient, as this health service reduces the cost of health care. Patients undergo fewer diagnostic and lab tests and less duration of stay than patients in the hospital, which explains the cost reduction [12]. A randomized controlled trial comparing hospital at-home programs with inpatient hospital settings reported a statistically significant overall cost reduction of 38%; fewer lab orders, imaging studies, and consultation orders; increased ambulation/activity levels; and decreased frequency of readmissions in the case of home hospital setting versus the usual hospital setting. The results of the study are summarized in Table 2 below [13].

**Table 2:** Showing outcomes comparison between home hospital and hospital setting

Outcomes compared	Home hospital (median per admission)	Hospital setting (median per admission)
Laboratory orders	3	15
Imaging orders	14%	44%
Consultation	2%	31%
Sedentary time	12%	23%
Lying down time	18%	55%
Readmission within 30 days	7%	23%

In the USA, where patient satisfaction is of utmost importance, HaH can aid in easing patients’ nervousness and offering a comfortable environment for better recovery. HaH care reduces readmissions and decreases the risks of life-threatening iatrogenic complications like nosocomial infections, delirium, and functional decline in elderly patients [14]. Health professionals in HaH provided a sense of normalcy in patients, especially in end-of-life care patients, as there was increased family engagement. There were also decreased anxiety levels in patients receiving care at home. The physician-patient relationship has also strengthened with this model of care. It also helped patients and physicians to work in a safe environment with improved patient-centered care during the Covid-19 pandemic. HaH has also decreased the burden of travel expenses for both patients and caregivers [15]. Recent advancements in-home monitoring has increased HaH’s competence to care for complex, high-acuity patients and increased the potential volume of patients safely treated at home [4]. For HaH programs that focused on continued care beyond the intervention, healthcare professionals helped integrate acute care into long-term management strategies [16].

The federal government considers most of the rural population in the USA medically underserved. Medical providers in rural settings do not want their patients sent away to the larger cities and health centers, which might be a long distance from home and a stressful journey by ambulance [17]. To meet the rising need for medical care in rural areas, recently, Ariadne Labs has partnered with the Thompson Family Foundation and three health systems, Blessing Health System of Quincy, IL, USA, Appalachian Regional Healthcare, Wetaskiwin Community Health Center, and Alberta Health Services, to conduct the first randomized controlled trial of the rural home hospital model. Under the model, patients are screened in the emergency department and, if they meet the criteria, are sent home with a tablet for telemedicine visits and appropriate digital health monitoring devices. A specially trained nurse sees the patient twice a day, and the physician addresses the patient once a day via telecommunication. The care team digitally monitors the health devices, and the patient can contact the care team anytime via the tablet [18].

### Challenges

One of the main challenges patients face in HaH models is medication management, since the “loose end” of this model is the at-home caregivers, who—despite being trained by in-home HaH staff at the outset of program enrollment—are not adept at administering medications or keeping track of which medications are for indication. Previous reviews of HaH programs have highlighted mistakes in medication administration, due to caregiver lack of familiarity with drug names and dosages. Beyond that, there are also logistical concerns with medication management in the HaH model—namely how prescribed medications are obtained and who administers them. There are several options for obtaining and administering drugs at home which have been developed. In general, how and where medications are obtained depends on the HaH program/platform being used, as well as on how a health system chooses to contract with local pharmacies. These medications prescribed by HaH clinicians could be obtained through a hospital pharmacy, a home health agency linked with the health system’s infusion pharmacy, or an independent contractual home infusion pharmacy. The medication reconciliation is performed by HaH staff at the time of intake, at the patient’s home [19].

Providing hospital-level services in the house also shifts a tremendous burden of care provision to medically untrained patients and/or their family members. Previous reviews have shown mistakes in medication administration, due to caregivers’ lack of familiarity with drug names and dosages. Empirical data suggests that at-home care patients suffer from malnutrition due to improper food selection and preparation [6]. Patients who lack caregiver presence at home are also a challenge for many; this is mitigated in hospitals where patients have the comfort of constant human contact from care professionals [9]. One of the major worries for HAH is the lack of doctors or nurses during emergency medical codes. From the physician’s perspective, the legal risk of medical malpractice is the main concern. Home care by nonprofessionals always has a probability of leading to bad outcomes, particularly since there is an in-built bias of choosing old patients. This makes physicians hesitant to refer patients to HaH care without due process to determine which care model fits a particular case. This causes an additional administrative burden in determining the right care model for a patient. Delays at the enrollment step cause poor throughput in emergency departments, and the time spent screening and enrolling patients into HaH is the time that could be better used in actually providing medical care to the patient. Due to the challenge of compensating care providers appropriately, much care coordination work goes uncompensated. Adding to this financial stress is the emotional toll from caring for senior at-risk patients, which can cause burnout in the care providers [6]. One of the essential components of good healthcare is coordination and teamwork among partners who specialize in different disciplines, and this can be very challenging in a hospital-at-home scenario due to its inherent asynchronous nature and the remote nature of things. Communication delays and a lack of robust referral systems can hamper the quality of care in this model [9].

There are several obstacles—among them being access to broadband Internet and experiencing slow Internet speeds—in accessing telehealth services to operate efficiently in rural HaH. One of the biggest barriers is access to broadband Internet and experiencing slow Internet speeds. Compared to their urban counterparts, rural individuals are nearly two times more likely to lack broadband access. In the absence of this infrastructure, it is incredibly difficult to deliver care across geographically large areas, where home visits are impossible. The shortage of healthcare professionals, who are the backbone of the HaH model, is another concern in rural areas [18].

Another challenge is the lack of insurance coverage for this model and the need for payers (commercial and governmental) to be flexible in their acceptance of this model. As previously mentioned, CMS has a standing waiver in place for HaH programs; commercial insurance plans, however, are varied in their approach to compensating health systems. Most payers utilize value-based reimbursement models, and we are unaware of any fee-for-service mechanisms in existing US HaH programs [6]. The shift towards value-based reimbursement sets up to be advantageous, however, particularly as the popularity and number of Medicare Advantage (MA) plans keep rising. Since MA plans are, themselves, dependent on maximizing quality and cost outcomes, reimbursement models that bend towards value, rather than fee-for-service, are favored. As alternative payment methods—which include bundled programs and Accountable Care Organizations—come to the forefront, HaH can make the case clinically and financially moving into the future. A strong foundation of experience with various funding structures—particularly in contract negotiation—will benefit health systems when considering HaH implementation [19].

## Conclusions

HaH represents a new-age healthcare delivery model that presents inherent benefits and challenges to patients, caregivers, providers, and health systems alike. Despite their aim to treat, hospitals still can lead to adverse events in some patients, especially the frail and elderly. With the HaH approach, patients benefit from receiving similar care provided at the hospital in the comfort and familiarity of their own homes, with the benefits of reducing costs and readmissions. With heavy reliance on family/in-home caregivers in the HaH model, strategies should be used to mitigate caregiver fatigue and burnout. Moving forward, systems choosing to implement HaH programs must ensure efforts focused on patient centeredness, individualization of care, and provider well-being are in place. This is achieved by providing shared decision-making involving caregivers and family members, as well as involving clinicians in implementation and quality oversight processes. Lastly, there is still a lack of a solid payment structure for HaH, and many health systems are left to approach HaH reimbursement on an individual level with commercial insurers. CMS policy, meanwhile, is currently dependent on the continuation of a waiver for HaH coverage, and there remains no formalization of HaH program recognition. We can conclude that for certain patients, HaH can actually prove to be a blessing for a rapid and uncomplicated recovery.

## Data Availability

Not applicable.

## References

[1] History. History hospital at home. https://www.hospitalathome.org/about-us/history.php. Published 2012. Accessed 15 Apr 2022.

[2] Leff B, Burton L, Guido S, Greenough WB, Steinwachs D, Burton JR (1999). Home hospital program: a pilot study. J Am Geriatr Soc..

[3] Foley OW, Ferris TG, Thompson RW (2021). Potential impact of hospital at home on postoperative readmissions. Am J Manag Care..

[4] Vaartio-Rajalin H, Ngoni K, Fagerström L (2019). Balancing between extremes-work in hospital-at-home. Nurs Open..

[5] Leff B, Burton L, Mader SL (2005). Hospital at home: feasibility and outcomes of a program to provide hospital-level care at home for acutely ill older patients. Ann Intern Med..

[6] Patel HY, West DJ (2021) Hospital at home: an evolving model for comprehensive healthcare. Allen Press. Retrieved April 16, 2022, from https://meridian.allenpress.com/innovationsjournals-JQSH/article/4/4/141/470333/Hospital-at-Home-An-Evolving-Model-for 10.36401/JQSH-21-4 PMC10229033 37261225

[7] Cheng J, Montalto M, Leff B (2009). Hospital at home. Clin Geriatr Med..

[8] Clarke DV, Newsam J, Olson DP, Adams D, Wolfe AJ, Fleisher LA. Acute hospital care at home: the CMS waiver experience. 10.1056/CAT.21.0338. Published December 7, 2021. Accessed 14 Apr 2022.

[9] Chua CMS, Ko SQ, Lai YF, Lim YW, Shorey S (2022). Perceptions of hospital-at-home among stakeholders: a meta-synthesis. J Gen Intern Med..

[10] Leff B, Montalto M (2004). Home hospital-toward a tighter definition. J Am Geriatr Soc..

[11] Hospital at home: patient care model of the future? https://www.todaysgeriatricmedicine.com/archive/0313p20.shtml. Accessed 11 Sept 2022

[12] Cryer L, Shannon SB, Van Amsterdam M, Leff B (2012). Costs for ‘hospital at home’ patients were 19 percent lower, with equal or better outcomes compared to similar inpatients. Health Aff (Millwood)..

[13] Levine DM, Ouchi K, Blanchfield B (2020). Hospital-level care at home for acutely ill adults: a randomized controlled trial. Ann Intern Med..

[14] Zolot J (2018). At-home hospital care reduces readmissions and length of stay, enhances patient satisfaction. Am J Nurs..

[15] Leff B (2009). Defining and disseminating the Hospital-at-Home model. CMAJ..

[16] Wilson A, Wynn A, Parker H (2002). Patient and carer satisfaction with ‘hospital at home’: quantitative and qualitative results from a randomised controlled trial. Br J Gen Pract..

[17] HealthLeaders. Can the hospital at home model help rural hospitals thrive? HealthLeadersMedia. https://www.healthleadersmedia.com/innovation/can-hospital-home-model-help-rural-hospitals-thrive. Accessed 11 Sept 2022.

[18] Rural Home Hospital. Ariadne Labs. https://www.ariadnelabs.org/home-hospital/rural-home-hospital/. Published November 18, 2021. Accessed 11 Sept 2022.

[19] “Hospital at home” programs improve outcomes, lower costs but face resistance from providers and payers. Commonwealth Fund. https://www.commonwealthfund.org/publications/newsletter-article/hospital-home-programs-improve-outcomes-lower-costs-face-resistance. Accessed 11 Sept 2022

